# Metastatic melanoma to the orbit masquerading as idiopathic orbital inflammation: a case report

**DOI:** 10.3389/fopht.2025.1678987

**Published:** 2025-11-07

**Authors:** Joshua J. Fernandes, Anna B. Sharabura, Matt M. Pfannenstiel, Jason A. Sokol

**Affiliations:** 1 Department of Ophthalmology, University of Kansas School of Medicine, Kansas City, KS, United States; 2 Department of Neurosurgery, University of Kansas School of Medicine, Kansas City, KS, United States

**Keywords:** idiopathic orbital inflammation, orbital melanoma, primary orbital melanoma, secondary orbital melanoma, case report

## Abstract

This case highlights an exceedingly rare presentation of secondary orbital melanoma and reviews the current literature on orbital melanoma.

**Case presentation:** A 39-year-old man was referred to our clinic after acute-onset decreased vision and left upper eyelid ptosis. Outside-hospital magnetic resonance imaging (MRI) showed an enhancing left superior orbital apex mass. The patient was initially treated for presumed idiopathic orbital inflammation without improvement. An orbital biopsy was performed, and the pathology showed secondary malignant melanoma. A computed tomography (CT) chest scan showed likely pulmonary metastases. Upon further questioning, the patient reported a history of an incompletely excised pigmented forearm lesion. The patient was treated systemically with nivolumab and ipilimumab.

**Conclusions and importance:** Despite its rarity, orbital melanoma should be considered in the differential diagnosis of patients with an orbital apex mass that does not respond to treatment for idiopathic orbital inflammation.

## Case presentation

**Figure 1:**
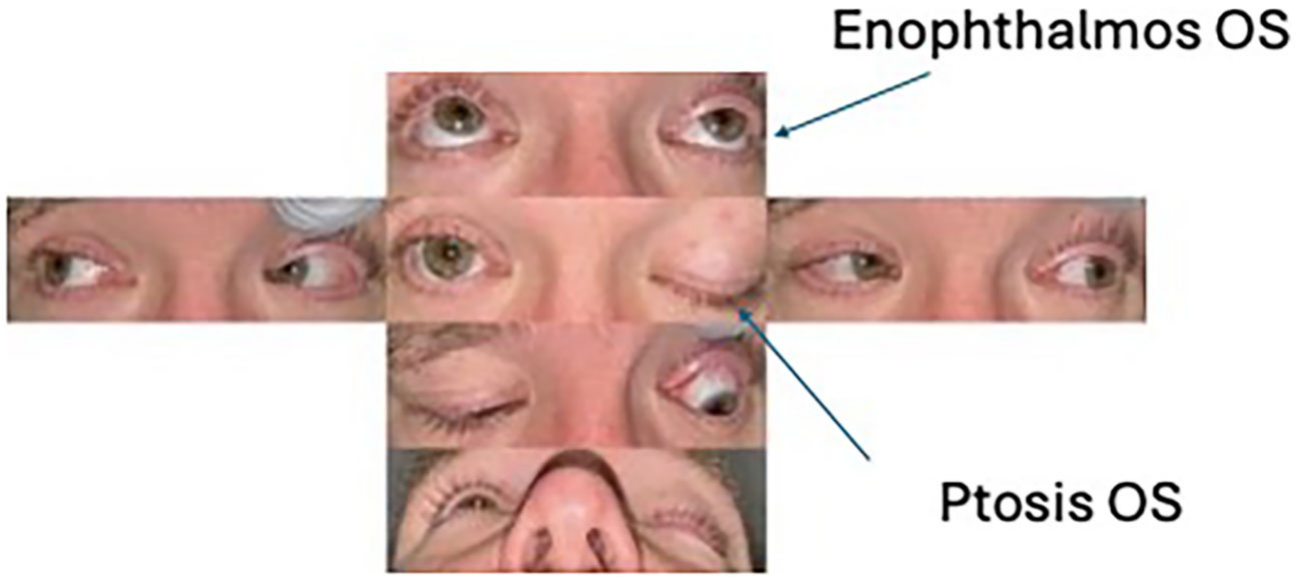
Motility images showing enophthalmos and ptosis OS without ophthalmoplegia.

**Figure 2:**
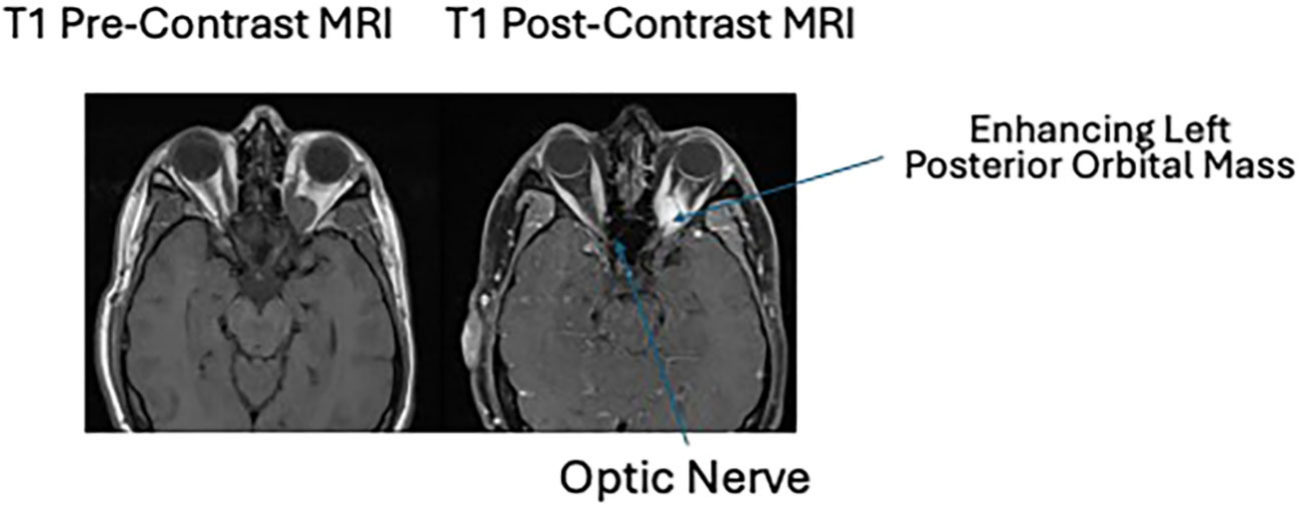
T1 pre- and post-contrast MRI imaging showing an enhancing left posterior orbital mass centered in the posterior intraconal fat just anterior to the optic nerve canal. This mass measures 2.3 × 1.5 × 1.3 cm, previously 2.3 × 1.4 × 1.3 cm when remeasured similarly. The lesion contains a single internal flow void. There is again inferior displacement of the optic nerve which is at least partially encased by the mass. At the orbital apex, the mass is inseparable from the extraocular muscles.

A 39-year-old man presented to an outside hospital for acute-onset left upper eyelid ptosis. The ophthalmic exam was notable for left-sided decreased visual acuity, upper eyelid ptosis, and optic disc edema (**Figure 1**). The MRI result revealed an enhancing mass in the left superior orbital apex (**Figure 2**). The laboratory studies were nonspecific with a slightly elevated erythrocyte sedimentation rate (ESR), C-reactive protein (CRP), and antinuclear antibodies (ANA). The patient was diagnosed with presumed idiopathic orbital inflammation, was treated with intravenous methylprednisolone, and was discharged on oral prednisone. However, with much surprise to the outside clinic, there was no improvement in his symptoms.

He was then referred to our oculoplastics clinic for evaluation. An orbital biopsy was performed through a superior medial lid crease incision. The surgical pathology showed malignant melanoma that was B-Raf proto-oncogene serine/threonine kinase (BRAF)-positive with a V600E mutation. The CT of chest/abdomen/pelvis for staging showed left chest nodal disease and likely pulmonary metastases.

Upon further questioning, the patient reported a history of a pigmented left forearm lesion that was removed 5 years prior to presentation. Outside pathology records were obtained, which showed a compound Spitz nevus extending to the skin shave biopsy peripheral margins with a maximum thickness of 2.95 mm. The lesion was reportedly treated with laser without complete excision.

## Discussion

The presence of primary orbital melanoma is exceptionally uncommon, representing less than 1% of optic melanomas (1). As of 2017, there were only 50 cases reported (2). In contrast, among secondary orbital malignancies, melanoma has been identified in 5%–10% of cases (3). The most common presenting symptom of primary orbital melanoma is proptosis (73%) (4). Other less common presenting symptoms include a decrease in visual acuity (32%), pain (14%), diplopia (15%), and a tangible mass (9%) (4). CT and MRI imaging are useful for both diagnosis and tracking the progression of orbital melanoma. The CT result demonstrates bony erosion with contrast enhancement, while the MRI result highlights homogeneous or heterogeneous enhancement that is similar to the primary lesion (2).

Periocular melanomas are one of the deadliest classes of melanomas; they most commonly appear within the uvea, less frequently within the conjunctiva, and are rarely observed within the orbit (5). Primary orbital melanomas are thought to originate from melanocytic cells of either the ciliary nerves or the leptomeninges. It is often associated with periocular pigmented changes, such as the Nevus of Ota, a typically benign lesion causing hyperpigmentation of the sclera (6).

Secondary orbital melanomas are typically associated with extra-scleral extensions of uveal melanomas and often present with a well-defined mass (7). These metastases tend to appear either within 1 year of the primary tumor or 3 years or more after a disease-free interval (3). For secondary melanomas, the varying time to presentation may be associated with the immune system’s control of tumor growth: a better prognosis may be connected with the presence of tumor-specific lymphocytes, such as tyrosinase-related protein (TRP)-1, TRP-2, melanoma antigen recognized by T cells (Melan-A/MART-1), glycoprotein (gp) 100, and tyrosinase (3).

Due to their similar histopathologic features, Spitzoid melanoma is often initially misdiagnosed as Spitz nevus, a benign melanocytic tumor (1, 8). In a study analyzing the histopathologic characteristics of 30 melanocytic lesions, 56.7% of cases were unable to be clearly identified as either Spitz nevus or melanoma (7). Both lesions have striking similarities in the mutations of their BRAF, neuroblastoma RAS viral oncogene homolog (NRAS), and Harvey rat sarcoma viral oncogene homolog (HRAS) genes, making it almost impossible to distinguish the two from each other in some cases (7). Recently, immunohistochemistry studies have been implemented to differentiate the two types of lesions: the Mindbomb Homolog-1 (MIB-1) proliferation marker, for example, has a nuclear proliferation index of 1.5% in Spitz nevus compared to 14.9% in Spitz melanoma (1). Comparative genomic hybridization studies have also demonstrated that the Spitz nevus tends to present with a gain of chromosome 11p that leads to an increased copy number of the HRAS allele, whereas melanomas lack this change (1).

In comparison to more conservative treatment methods, surgical treatment of secondary orbital melanomas combined with accessory radiotherapy and/or chemotherapeutic regimens has not been shown to increase a patient’s survival. Orbital exenteration has been shown to limit the localized spread of orbital metastases (3). However, more recently, there has been increased consideration toward the use of immunotherapy to treat orbital melanomas. Specific targets include anti-programmed cell death protein-1 (PD-1), anti-programmed cell death ligand-1 (PDL-1), and anti-cytotoxic t-lymphocyte-associated protein-4 (CTLA-4) inhibitors. In particular, nivolumab, pembrolizumab, and ipilimumab are effective against cutaneous and conjunctival melanomas (9, 10). Furthermore, mutations in two genes of the mitogen-activated protein kinase, BRAF and NRAS, have been implicated in 80% of melanoma cases (11). In a recent study, combined immune therapy with ipilimumab and nivolumab in patients with advanced melanoma led to an 80% reduction in tumor burden, compared to a 20% reduction in patients who were treated with either drug alone (11).

Idiopathic orbital inflammation (IOI) is a benign heterogeneous inflammatory condition without a definitive origin or cause that accounts for 8%–10% of mass lesions in the orbit (12). Diagnosis is based on a tissue biopsy with nonspecific histologic findings of a polymorphous admixture of non-atypical lymphocytes, eosinophils, macrophages, and neutrophils that respond well to systemic corticosteroids (13). The two leading hypotheses on IOI include autoantibodies to ocular muscle antigens and viral infection, though no definitive cause has been isolated (14). A related condition of orbital cellulitis, often caused by auto-infarction and tumor necrosis, can precede a diagnosis of melanoma. In particular, a study on the prevalence of uveal melanoma found that 47% of patients, who were later diagnosed with uveal melanoma, presented with non-specific orbital cellulitis (15).

## Patient update

The patient continues monitoring of his stage IV (cN3b, pM1c) BRAF V600E + metastatic melanoma of the skin with oncology during his participation in a clinical trial (ipilimumab and nivolumab). He has had improvement in the size and degree of enhancement of the left orbital mass, although he has mild optic nerve atrophy seen on CT orbits. He does not have intracranial metastatic disease, and the axillary lymphadenopathy and left subpectoral metastasis are stable. The oncology team believes that due to the positive response to immunotherapy, the primary site was cutaneous. His only side effects have been the whitening of his hair and beard. His liver function tests (LFTs) have been elevated and are monitored closely.

## Conclusion

Despite its rarity, orbital melanoma should be considered in the differential diagnosis of patients with an orbital apex mass that does not respond to treatment for idiopathic orbital inflammation. To advance diagnostic awareness, future studies could investigate orbital melanoma to further understand its varied presentation and pathophysiological characteristics.

## Data Availability

The original contributions presented in the study are included in the article/supplementary material. Further inquiries can be directed to the corresponding author.
